# Media Composition Effects on Hairy Root Biomass and Tetrandrine Production in *Stephania tetrandra*

**DOI:** 10.3390/molecules30081859

**Published:** 2025-04-21

**Authors:** Chia-Hung Kuo, Hsuan-Chieh Liu, Parushi Nargotra, Hsiao-Sung Chan, Hsin-Der Shih, Yung-Chuan Liu

**Affiliations:** 1 Department of Seafood Science, National Kaohsiung University of Science and Technology, Kaohsiung 811, Taiwan; kuoch@nkust.edu.tw (C.-H.K.); parushi11nargotra@gmail.com (P.N.); 2 Department of Chemical Engineering, National Chung Hsing University, Taichung 402, Taiwan; ohohpigjay@gmail.com; 3 Central Region Campus, Industrial Technology Research Institute (ITRI), Nantou 540, Taiwan; 4 Department of Applied Chemistry, Chaoyang University of Technology, Taichung 413, Taiwan; hsiaosungchan@gmail.com; 5 Plant Pathology Division, Taiwan Agricultural Research Institute, Ministry of Agriculture, Taichung 413, Taiwan; tedshih@tari.gov.tw

**Keywords:** *Stephania tetrandra* S. MOORE, hairy roots, central composite design, optimization, tetrandrine

## Abstract

*Stephania tetrandra* S. MOORE, a medicinal plant, is recognized for tetrandrine production, which is extensively accepted for its therapeutic benefits. However, the slow growth of *S. tetrandra* limits tetrandrine’s availability, which can be addressed by in vitro hairy root cultivation using *Rhizobium rhizogenes* and optimization of medium components. The present study attempted the three-step optimization of three components of woody plant medium (ammonium nitrate, calcium nitrate, and sucrose), including two-level factorial design, path of steepest ascent (PSA), and central composite design (CCD) to obtain high hairy root biomass and tetrandrine production. The CCD-based optimization for enhanced hairy root biomass resulted in a hairy root biomass of 9.75 g dw/L at optimal concentrations of ammonium nitrate (NH_4_NO_3_), calcium nitrate (Ca(NO_3_)_2_), and sucrose of 631.96 mg/L, 651 mg/L, and 41.35 g/L, respectively. The optimal concentration of 550.31 mg/L, 862.88 mg/L, and 25.89 g/L for NH_4_NO_3_, Ca(NO_3_)_2_, and sucrose, respectively, obtained after CCD analysis for enhanced tetrandrine production led to the maximum tetrandrine of 70.48 mg/L. Medium optimization resulted in a 1.47-fold increase in hairy root biomass and a 1.37-fold increase in tetrandrine production under individually optimized conditions. The present study findings confirmed the important role of process optimization for enhanced product yield.

## 1. Introduction

World health is being impacted by the growing global interest in medicinal plants. Plant-based herbal (traditional) medicine has been a cornerstone of basic healthcare systems worldwide. For millennia, this ancient medical system has been instrumental in preserving the health of numerous individuals, particularly in Asian communities [1]. Due to the recent embrace of the “return to nature” initiative and expanding comprehension of the pharmacological attributes of medicinal plants, herbal medicine is starting to gain acknowledgment as a viable and effective option for the administration of future healthcare [2]. Currently, billions of individuals worldwide consume food, medications, or supplements containing traditional herbal medicine on a daily basis [3]. Both developed and developing countries are seeing an increase in the knowledge and progress of the medical effects of plants. Medicinal plants have been the mainstay of complementary treatment and have become a major source for developing novel pharmaceuticals. The increased reliance on medicinal plants for addressing illnesses stems from their reputation as safe and effective therapies, presenting minimal adverse reactions and cost-effectiveness [4]. Plant metabolites are acknowledged for demonstrating various properties, including antidiabetic, antitumor, anti-inflammatory, anti-rheumatoid arthritis, antioxidant, and anti-Parkinson’s effects [5,6,7].

Many plants have been explored for their medicinal properties such as *Cannabis Sativa*, *Fissistigma oldhamii*, *Chamaecrista nigricans*, *Senna occidentalis*, *Vernonia amygdalina*, *Abrus precatorius*, *Aloe nobilis*, *Taxus* sp., *Acalypha godseffiana*, *Azadirachta indica*, *Amaranthus hybridus*, *Aloe vera*, etc. [6,7,8,9,10,11]. *Stephania tetrandra* S. MOORE, also known as Fen Fang Ji, is one such plant that is extensively acknowledged for its therapeutic importance, specifically in Japan, China, and South Korea [12]. This herbaceous vine has anti-inflammatory, neuroprotective, and antiviral attributes and is recognized for treating conditions such as cancer, edema, rheumatism, malaria, rheumatoid arthritis, hypertension, and hyperglycemia [13,14]. Tetrandrine, one of the primary benzylisoquinoline-type alkaloids produced by *S. tetrandra*, stands out for its diverse and remarkable biological effects, including antitumor, anti-microbial, anti-inflammatory, neuroprotective against the Ebola virus and COVID-19 [15]. Research conducted both in vitro and in vivo has demonstrated that it functions as a powerful calcium channel blocker and is an effective anticancer drug [16].

Despite its therapeutic benefits, tetrandrine’s availability is limited by the slow growth and diminishing wild resources of *S. tetrandra*, driving up demand and prices of *S. tetrandra* [17]. These limitations can be addressed by using biotechnological methods, such as in vitro cultivation through *Rhizobium rhizogenes*-induced hairy root production. The productivity of tetrandrine can also be enhanced by optimization of the medium components for enhanced hairy root biomass and/or tetrandrine production. Optimization of any process offers a chance to boost production yield while simultaneously making the process economical [18,19]. Statistical optimization using response surface methodology (RSM) is superior to the one-variable-at-a-time (OVAT) method, as it allows for the simultaneous assessment of the combined effects of multiple factors and their interactions [20]. In contrast, OVAT examines each factor independently while keeping others constant, thereby overlooking potential inter-variable interactions. In addition, RSM typically requires fewer experimental runs than OVAT, achieving more reliable optimization outcomes [21]. This enables a more thorough and effective optimization of complex systems involving multiple interacting variables. Nevertheless, OVAT remains useful for the initial screening of significant medium components, which can then be further refined using RSM to determine optimal concentrations and evaluate interaction effects [22]. A study reported a 1.49-fold increase in resveratrol production (133.53 µg/mL) from *Arcopilus aureus* after RSM-based optimization of physicochemical and nutritional parameters compared to a 1.23-fold increase using the OVAT approach [23].

Considering the aforementioned factors, the present study reports the optimization of medium components, including NH_4_NO_3_, Ca(NO_3_)_2_, and sucrose, for enhanced *S. tetrandra* hairy root biomass and tetrandrine production. The production of hairy roots was carried out from the leaves of *S. tetrandra* using *Rhizobium rhizogenes* ATCC15834 [24]. The optimization was executed in three consecutive steps involving two-level factorial design (FFD), path of steepest ascent (PSA), and central composite design (CCD) methods to attain the optimal concentrations of medium components. The current work is a follow-up to a previously published study on tetrandrine production using the hairy root culture method of *Stephania tetrandra* S. MOORE [24]. The response surface methodology approach was used in this study to further optimize the composition of the woody plant medium, which was identified as the best base medium for maximizing biomass and tetrandrine production. This optimization methodology differs from the one-factor-at-a-time approach used in the previously reported study [24]. The objective of this study was to optimize the culture medium components to enhance both hairy root biomass and tetrandrine production in *Stephania tetrandra*. A two-level factorial design was initially employed to identify the significant variables influencing both responses simultaneously. This design generated separate predictive models for biomass and tetrandrine yield. Based on these models, the optimization process was then carried out independently for each hairy root biomass and tetrandrine production, following separate paths of steepest ascent and response surface methodology to account for the differing physiological requirements underlying root proliferation and alkaloid biosynthesis.

## 2. Results and Discussion

### 2.1. Two-Level Factorial Experimental Design

**Figure 1:**
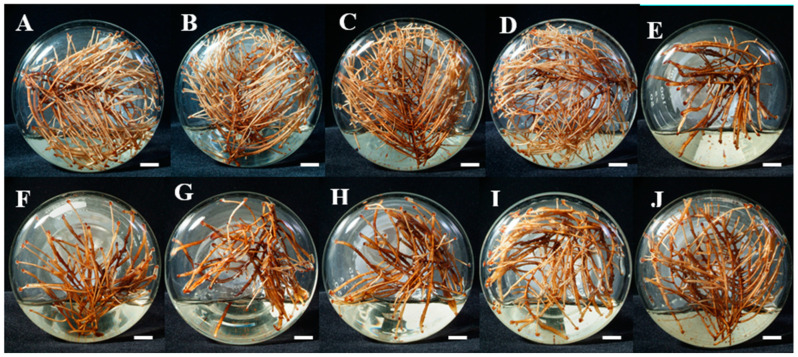
Cultivation of *S. tetrandra* hairy roots based on experiments generated by a two-level factorial design of three medium components (ammonium nitrate, 500 mg/L, calcium nitrate, 756 mg/L, and sucrose, 40 g/L) for 5 weeks. (**A**)—experiment 1, (**B**)—experiment 2, (**C**)—experiment 3, (**D**)—experiment 4, (**E**)—experiment 5, (**F**)—experiment 6, (**G**)—experiment 7, (**H**)—experiment 8, (**I**)—experiment 9, (**J**)—experiment 10. Bar = 1 cm.

The use of experimental factorial design is widely recognized as a conventional method for identifying the optimal parameters needed to achieve high product yield [25]. Factorial design involves setting diverse independent variables, comprising both quantitative and qualitative factors, at predetermined levels, followed by their examination in a predefined set of experiments. This methodology enables a direct evaluation of how these variables affect dependent variables [26]. A two-level FFD of three factors, including Ca(NO_3_)_2_ (756 mg/L), sucrose (40 g/L), and NH_4_NO_3_ (500 mg/L), was conducted. An FFD was employed to determine the positive direction (or slope) for every variable. The results are shown in Table 1 and Figure 1. According to the results, maximum tetrandrine production (58 mg/L) was obtained in the fourth experiment of culture medium formulations with 600 mg/L ammonium nitrate, 956 mg/L calcium nitrate, and 30 g/L sucrose. It was also discovered that the production of tetrandrine was higher at a 30 g/L sucrose concentration than at 40 and 50 g/L sucrose. However, the hairy root biomass (8.92 g dw/L) was maximum at a sucrose concentration of 40 g/L. After performing regression analysis and calculation of the hairy root biomass (Y_1_) and tetrandrine production (Y_2_) obtained from FFD, three different regression equations were obtained. The coefficient before each factor in the equation (Equation (1)) represented the impact of that factor on the result.

The regression equation calculated from the hairy root mass (Y_1_) is as follows:

Y_1_ = 7.82 + 0.32X_1_ − 0.15X_2_ + 0.03X_3_ (1)

According to the equation, an increase in the concentration of ammonium nitrate (X_1_) and sucrose (X_3_) led to an increase in hairy root biomass, indicating the positive impact of these two factors, whereas calcium nitrate (X_2_) showed a negative effect on the biomass. As compared to NH_4_NO_3_, sucrose had little impact on the biomass.

The regression formula equation (Equation (2)) for tetrandrine production (Y_2_) is as follows:

Y_2_ = 37.51 + 3.09X_1_ + 3.98X_2_ − 11.42X_3_ (2)

Like regression equation Y_2_, a positive effect of ammonium nitrate (X1) and calcium nitrate was observed on the tetrandrine yield, whereas sucrose had a negative effect. On comparing the results of the one-factor-at-a-time approach (previous study) and the FFD, the effects of the three factors were consistent. It was evident from the results that the concentrations of ammonium nitrate and sucrose were crucial for improved hairy root biomass, whereas tetrandrine production is directly correlated to the increase in the concentration of ammonium nitrate and calcium nitrate.

### 2.2. Path of Steepest Ascent Analysis for Hairy Root Biomass

**Figure 2:**
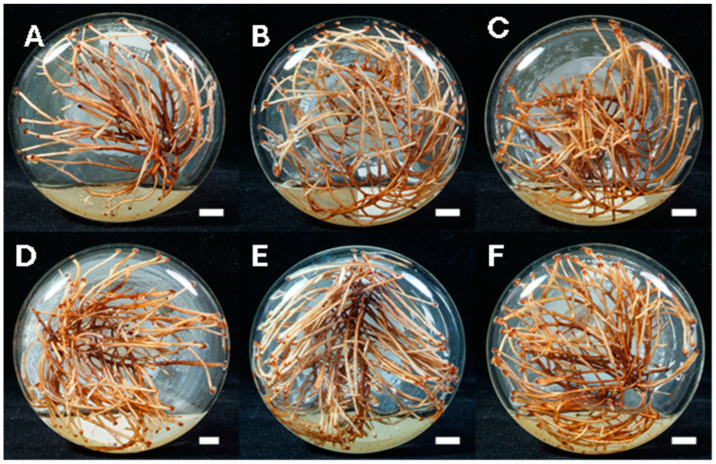
Cultivation of *S. tetrandra* hairy roots for 5 weeks based on experiments generated by steepest ascent path analysis of three medium components for hairy root biomass. (**A**)—experiment 1, (**B**)—experiment 2, (**C**)—experiment 3, (**D**)—experiment 4, (**E**)—experiment 5, (**F**)—experiment 6. Bar = 1 cm.

The PSA approach was applied to ascertain the most favorable direction of concentration of the studied variables as per the results of FFD. This approach entails moving progressively in the direction of the ideal concentration location. In accordance with the regression equation derived from the FFD, a high value is chosen for a variable when its effect is positive, whereas a low value is selected when the effect is negative [19]. In the current experiment, the regression equation (Y_1_) of hairy root biomass generated by FFD analysis was selected for the PSA analysis. In the six experiments, each factor was either increased (ammonium nitrate, 32 mg/L, and sucrose, 0.3 g/L) or decreased (calcium nitrate, −30 mg/L) as per the equation. The results after five weeks of cultivation are represented in Table 2 and Figure 2. The results indicated that the amount of hairy root biomass either increased or decreased in response to changes in the concentrations of the three components in the culture medium. Experiment 5 yielded the highest hairy root biomass (9.33 g dw/L), achieved with concentrations of 628 mg/L for ammonium nitrate, 636 mg/L for calcium nitrate, and 41.2 g/L for sucrose. The decrease in hairy root biomass amount was observed as the concentration moved along the steep ascending path. Thus, it was inferred that the concentration of each component in the fifth experimental group should be situated closest to the extreme value zone. Consequently, in the subsequent central composite design analysis, the concentration levels from the fifth group of culture medium were adopted as a fresh initial point to determine the position of the extreme point.

### 2.3. Central Composite Design Analysis for Hairy Root Biomass

The optimal concentrations of ammonium nitrate (628 mg/L), calcium nitrate (636 mg/L), and sucrose (41.2 g/L), determined from the steep rise path experiment results, served as the starting point for the CCD analysis to locate the location of the extreme point more precisely. The results of the CCD analysis for enhanced hairy root biomass are shown in Table 3. The relative impact of the factors on the hairy root biomass was identified via the coded equation by comparing the factor coefficients. The regression equation (Equation (3)) generated by the model indicated the positive impact of calcium nitrate and sucrose on the hairy root biomass.

Biomass (g dw/L) = 7.73 − 0.0983X_1_ + 0.5209X_2_ + 0.3487X_3_ + 0.1150X_1_X_2_ + 0.1125X_1_X_3_ + 0.1750X_2_X_3_ − 0.2607X_1_^2^ + 0.5826X_2_^2^ − 0.0662X_3_^2^ (3)

The results of the analysis of variance (ANOVA) experiment are provided in Table S1. According to the ANOVA analysis, the model was significant, with a model F-value of 9.61 and probability < 0.05. Independent factors calcium nitrate and sucrose had a significant positive impact on the hairy root biomass production. The predicted R^2^ of 0.6768 was in logical agreement with the adjusted R^2^ of 0.8379, showing a difference of less than 0.2 and, therefore, indicating the strength of the model. Moreover, the adequate precision of 11.496, which measures the signal-to-noise ratio, indicated an adequate signal. The predicted versus experimental (actual) plot showed a good agreement between experimental and model-predicted values, indicating the validity of the developed RSM model (Figure S1). The minimal deviation of the data points from the diagonal line confirmed the model’s predictive power for optimizing hairy root biomass. This supported the robustness of the mathematical model used in this study. In this case, X_2_ (calcium nitrate), X_3_ (sucrose), and X_2_^2^ were significant model terms with *p*-values less than 0.05. Calcium is an essential macronutrient that has a variety of functions in plants. It maintains the rigidity of cell walls, upregulates transcriptional regulators of secondary metabolites, functions as an intracellular second messenger, and is a component of membranes and cell walls [27,28]. In plant culture, sucrose is also valuable for regulating many genes and stress responses, maintaining cellular osmotic potential, impacting growth, development, differentiation, and metabolic pathways, and being essential for metabolite production. Its concentration has been shown to have a discernible effect on growth rates and secondary metabolites’ yield [29].

**Figure 3:**
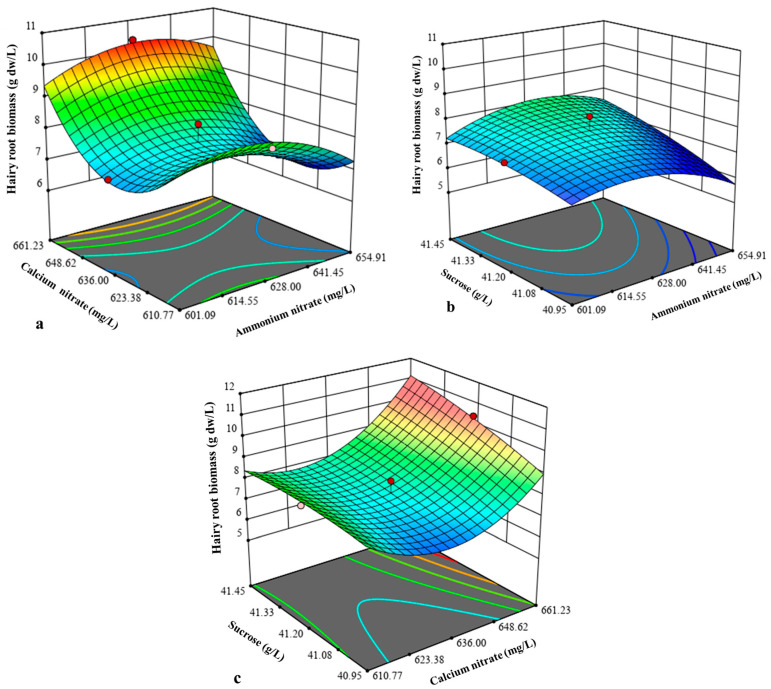
Response surface plot showing the interaction between ammonium nitrate and calcium nitrate (**a**), ammonium nitrate and sucrose (**b**), and calcium nitrate and sucrose (**c**) for *S. tetrandra* hairy root biomass.

The influence of interactions between the independent variables on hairy root biomass was depicted by three-dimensional response surface plots (Figure 3). The impact of all three interactions, i.e., ammonium nitrate and calcium nitrate (Figure 3a), ammonium nitrate and sucrose (Figure 3b), and calcium nitrate and sucrose (Figure 3c), was positive on the hairy root biomass production but insignificant, besides CCD analysis verification. During the validation experiment, the experiment was conducted at the optimal concentration of NH_4_NO_3_ (631.96 mg/L), Ca(NO_3_)_2_ (651 mg/L), and sucrose (41.35 g/L) generated by the model with a predicted biomass yield of 9.3 g dw/L. The experimental hairy root biomass obtained was 9.75 g dw/L, which was in close proximity to the predicted value. The optimization resulted in a 1.47-fold enhancement in hairy root biomass as compared to control hairy root biomass (6.61 g dw/L) cultivated in WPM medium containing 400 mg/L NH_4_NO_3_, 556 mg/L Ca(NO_3_)_2_, and 30 g/L sucrose. This result confirmed that the hairy root culture medium designed by the CCD improved the hairy root growth quality. In addition, the results found that the yield of tetrandrine did not increase. It can be speculated that even after five weeks of cultivation, the nutrients in the culture medium were not depleted, and the growth stasis period was not reached. Therefore, extending the cultivation time may aid in achieving better results with respect to tetrandrine production. Similar to our findings, CCD-based optimization also enhanced hairy root biomass from *Astragalus membranaceus*. An optimal hairy root biomass of 15.79 g/L dry weight was achieved at an inoculum size of 1.54%, culture temperature of 27.8 °C, sucrose concentration of 3.24%, and harvest time of 36 days [30].

### 2.4. Path of Steepest Ascent Analysis for Tetrandrine Production

**Figure 4:**
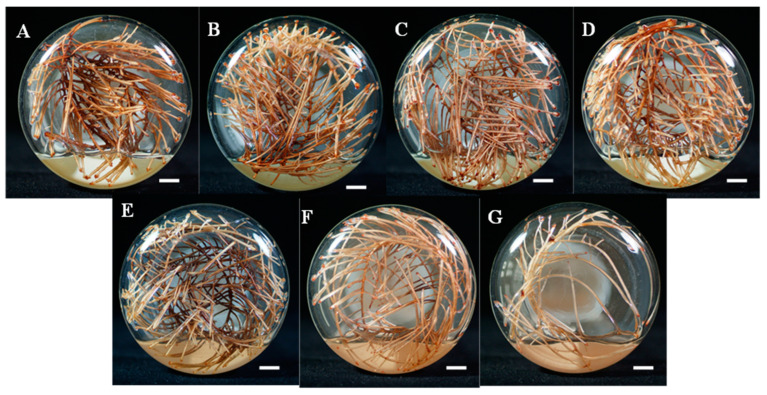
Cultivation of *S. tetrandra* hairy roots for 5 weeks based on experiments generated by steepest ascent path analysis of three medium components for tetrandrine production. (**A**)—experiment 1, (**B**)—experiment 2, (**C**)—experiment 3, (**D**)—experiment 4, (**E**)—experiment 5, (**F**)—experiment 6, (**G**)—experiment 7. Bar = 1 cm.

According to the results obtained from the FFD analysis, the regression equation generated based on tetrandrine production (Y_2_) was selected, and only factor ammonium nitrate (X_1_) and calcium nitrate (X_2_) had a positive impact on tetrandrine production. The coefficients in the regression equation for tetrandrine production for the respective factors were multiplied by the unit of that factor in the FFD to obtain a new set of experiments. The base point for the PSA method is established using the center point of the FFD experiment (Table 4). Seven experiments were generated to determine the distance required for each factor to increase (ammonium nitrate 15.45 mg/L, calcium nitrate 39.8 mg/L) or decrease at each step (sucrose −5.71 g/L). After five weeks of cultivation, the hairy root biomass and yield were analyzed. It was observed that as the concentration of the three components in the culture medium increased or decreased, the production of tetrandrine in the hairy roots of *S. tetrandra* also increased gradually (Table 4 and Figure 4). In experiment 4, the highest tetrandrine production (65.22 mg/L) was achieved at ammonium nitrate concentrations of 546.35 mg/L, calcium nitrate concentration of 875.4 mg/L, and sucrose concentration of 22.87 g/L, respectively. After experiment 4 of the steep ascending path, even when the concentration of ammonium nitrate and calcium nitrate was increased and sucrose was decreased, the biomass of tetrandrine hairy roots and the production of tetrandrine also decreased accordingly (Table 4). Therefore, it was speculated that the concentration of each component in the fourth group of culture medium was the closest and the extreme value zone is within the range. Therefore, for the central composite analysis, the concentration of three components as per experiment 4 of PSA was used as a new starting point to find the location of the extreme point. The combination of steepest ascent test analysis and RSM has been utilized to optimize the medium components K_2_HPO_4_, soybean cake powder, and soluble starch for enhancing the mycelial growth and metabolite production of *Streptomyces alfalfae* XN-04. Consistent with our research, an initial increase in mycelial growth was observed, followed by a subsequent decrease. The concentration of medium components yielding the maximum yield was selected for further optimization [31].

### 2.5. Central Composite Design Analysis for Tetrandrine Production

In the hairy root biomass optimization study, the optimization of concentration media components for the improved quality of *S. tetrandra* hairy root biomass was also attempted. The optimal concentration of ammonium nitrate (631.96 mg/L), calcium nitrate (651 mg/L), and sucrose (41.35 g/L) improved the hairy root biomass to 9.75 g dw/L from 6.61 g/L but did not increase the tetrandrine production (48.49 mg/L). The incomplete utilization of medium nutrients and failure to enter the growth stasis phase for tetrandrine synthesis was postulated as the causes. As a result, this study tried to optimize the media’s constituents for increased tetrandrine production. With the optimal concentrations of sucrose, ammonium nitrate, and calcium nitrate for enhanced tetrandrine production aligning with experiment 4 of the PSA, the central composite design was recentered at the following values: 546.35 mg/L of ammonium nitrate, 875.40 mg/L of calcium nitrate, and 22.87 g/L of sucrose. The result of 16 experiments generated by central composite design is shown in Table 5. The impact of ammonium nitrate (X_1_) and sucrose (X_3_) was significant on tetrandrine production, as depicted by the regression Equation (4).

Tetrandrine production (mg/L) = 66.62 + 2.06X_1_ − 1.64X_2_ + 6.01X_3_ − 1.55X_1_X_2_ − 0.1700X_1_X_3_ + 1.42X_2_X_3_ − 0.4889X_1_^2^ − 0.9857X_2_^2^ − 4.38X_3_^2^ (4)

The data from experiments 2, 3, 15, and 16 suggested an influence of Ca(NO_3_)_2_ concentration on the production of tetrandrine, as indicated by the varying yields of tetrandrine under different experimental conditions (Table 5). The data from experiments 2 and 16, where the concentration of calcium nitrate (895.30 mg/L) remains constant, show differing tetrandrine yields (50.68 ± 1.77 mg/L and 66.84 ± 5.19 mg/L, respectively). While calcium nitrate plays a role in plant secondary metabolism, other factors, such as the concentration of ammonium nitrate and sucrose, also influence tetrandrine production. In experiment 16, the increase in ammonium nitrate (554.07 mg/L) and sucrose (28.58 g/L) concentrations compared to experiment 2 might provide the necessary carbon and nitrogen sources to maintain cellular functions. Calcium plays a critical role in various metabolic processes, including those related to secondary metabolism and stress responses in plants or hairy root cultures. Calcium ions may act as second messengers in various biochemical pathways. This is particularly relevant in the context of alkaloid biosynthesis, as calcium has been implicated in regulating the expression of enzymes involved in the production of secondary metabolites [32,33,34,35].

**Figure 5:**
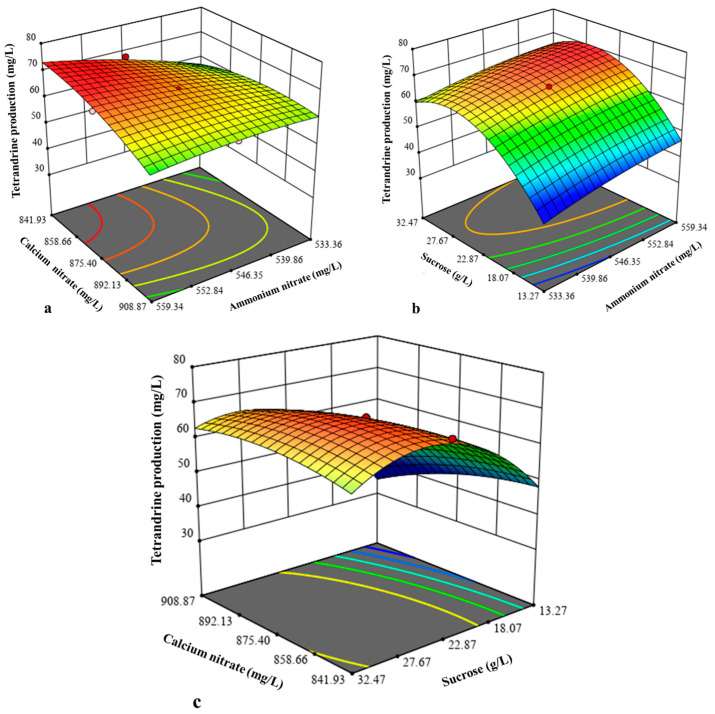
Response surface plot showing the interaction between ammonium nitrate and calcium nitrate (**a**), ammonium nitrate and sucrose (**b**), and calcium nitrate and sucrose (**c**) for the production of tetrandrine.

ANOVA indicated the fitness of the model, and the model was significant with a *p*-value of 0.0007 and a model F-value of 21.58 (Table S2). The predicted R-squared (0.7725) was in reasonable agreement with the adjusted R-squared (0.9251), and an adequate precision ratio of 16.153 indicated an adequate signal, further implying a good relation between predicted and experimental values. With *p*-values less than 0.05, variables X_1_ (ammonium nitrate), X_2_ (calcium nitrate), X_3_ (sucrose), and X_3_^2^ were significant model terms. The plot of experimental values versus predicted values also demonstrated a strong correlation between model predictions and experimental results. The proximity of data points to the diagonal line indicated high model accuracy, validating the effectiveness of the response surface methodology employed in optimizing culture conditions (Figure S2). Like calcium and sucrose, ammonium nitrate is also vital for plant growth. Environmental conditions, developmental stages, and plant species affect the optimal ammonium-to-nitrate ratio. A 50:50 ratio may be ideal for plant growth since ammonium promotes aboveground growth, and nitrate, while beneficial for root development, might have adverse effects on aboveground portions when present in high amounts. Furthermore, nitrogen sources such as ammonium nitrate widely affect root growth, proliferation of cells, and metabolite synthesis in plant cell cultures [36,37]. The impact of interactions between the three factors, i.e., ammonium nitrate and calcium nitrate (X_1_X_2_), ammonium nitrate and sucrose (X_1_X_3_), and calcium nitrate and sucrose (X_2_X_3_) on the tetrandrine production is shown in Figure 5. The interaction between ammonium nitrate and calcium nitrate (Figure 5a) and ammonium nitrate and sucrose (Figure 5b) had a negative but insignificant effect on the tetrandrine production. However, the interaction between calcium nitrate and sucrose (Figure 5c) showed a positive impact (insignificant) on tetrandrine production, as suggested by the model. The impact of three interactions was also evidenced by the regression Equation (4).

The CCD was validated by conducting the experiment as per the optimal concentration of ammonium nitrate, calcium nitrate, and sucrose obtained from the Design Expert model were 550.31 mg/L, 862.88 mg/L, and 25.89 g/L, respectively, predicting maximum tetrandrine production of 70.12 mg/L. The experimental tetrandrine production of 70.48 mg/L was obtained, which was in reasonably good agreement with the predicted value and was in the design space. An increase of 1.37-fold in tetrandrine production was observed compared to the control unoptimized WPM medium (51.35 mg/L). Moreover, it was interesting to note that even though the tetrandrine production increased under optimal conditions, the root biomass was less (7.89 g dw/L) as compared to the 9.75 g dw/L obtained during hairy root biomass optimization. While biomass is typically associated with optimal growth, secondary metabolite production, such as tetrandrine, is often triggered under stressful conditions, such as high calcium or nitrate concentrations. Recent research highlights that excessive calcium in the plant medium can inhibit the growth of hairy root biomass as it may disrupt cellular processes such as cell division or water uptake. Specifically, studies have demonstrated that while optimal levels of calcium can enhance plant growth, an overabundance can lead to a significant decrease in biomass. For example, a study on poplar seedlings indicated that increasing calcium concentrations initially boosted biomass, but excessive calcium did not result in further biomass increase, suggesting a plateau and eventual negative impact on growth [38]. Moreover, high concentrations of calcium ions can induce salt stress, which may in turn stimulate secondary metabolite production [33]. Since calcium ions also act as secondary messengers in signal transduction pathways, they may potentially activate calcium-dependent protein kinases (CDPKs), which may regulate the biosynthesis of secondary metabolites [34]. Similarly, even though nitrate is an essential nutrient, high levels have been shown to negatively affect biomass in certain plant species, such as *Atropa belladonna* and *Vitis vinifera* [39,40]. Additionally, secondary metabolite production is often enhanced under nutrient-limited or stressful conditions, which can trigger defense responses in the plant and result in the accumulation of metabolites like tetrandrine. This may explain why, in this study, the increased tetrandrine production did not correlate directly with biomass growth. When plant cells experience stress, they often shift energy away from primary growth pathways to secondary metabolic pathways, which is commonly seen in secondary metabolites [41,42]. Therefore, although higher biomass might be expected to correlate with more secondary metabolites, under certain conditions, such as nutrient excess or stress, the plant may prioritize defense mechanisms over its growth, resulting in increased metabolite production but reduced biomass. CCD has been used for the improvement in the production of plant metabolites. A study reported that the CCD-based optimization of methyl jasmonate concentration (100–400 μM) and exposure duration (12–48 h) enhanced the production of isoflavonoid from *Astragalus membranaceus* hairy roots by 9.71-fold (2250.10 ± 71.88 μg/g) as compared to the control [43]. In another study, the production of pharmacologically active alkaloids and flavonoids from the hairy roots of *Isatis tinctoria* L. was optimized using CCD, which resulted in a 5.89- and 11.21-fold increase in alkaloids and flavonoids, respectively, as compared to the control [44]. The results of the present study clearly explain the crucial role of optimization in producing value-added products. Optimization not only enhances the yield but also aids in making the production process economically viable [45].

## 3. Materials and Methods

### 3.1. Chemicals and Plant and Bacterial Strain

*Rhizobium rhizogenes* ATCC15834 (BCRC15010) was procured from the Bioresource Collection and Research Centre (BCRC) of the Food Industry Research and Development Institute, Hsinchu, Taiwan. The dried medicinal components of *Stephania tetrandra* that are commercially available and tetrandrine were acquired from a traditional Chinese medicine store in Taichung, Taiwan and Sigma (St. Louis, MO, USA), respectively. Sterile *Stephania tetrandra* S. Moore (*S. tetrandra*) was provided by Prof. Chao-Lin Kuo of China Medical University, Taichung, Taiwan.

### 3.2. Establishment and Selection of Hairy Roots from S. tetrandra S. MOORE

According to the previous study [24], hairy root line HR10-1, which was infected by *Rhizobium rhizogenes* ATCC15834 (BCRC15010), exhibited high hairy root growth quality and tetrandrine content. Therefore, in the present study, hairy root line HR10-1 was selected for further analysis. HR10-1 was previously grown for five weeks in a modified liquid woody plant medium (WPM) having Ca(NO_3_)_2_ (556 mg/L) and NH_4_NO_3_ (400 mg/L) supplemented with 3% sucrose and 0.3% gelrite (pH of 5.2 ± 0.1). The medium was kept dark and at a constant 25 ± 2 °C with 80 rpm of shaking. The medium composition for improved hairy root biomass and tetrandrine production was optimized using RSM.

### 3.3. Two-Level Factorial Experimental Design and Path of Steepest Ascent Design

The concentration of Ca(NO_3_)_2_, NH_4_NO_3_, and sucrose in the WPM medium was optimized using RSM. The two-level FFD, PSA, and CCD methods were carried out sequentially, and the experimental results were obtained. The regression model was used to explore the location of the extreme value to perform an optimization of the medium components to obtain the optimal concentration of the medium components for enhanced hairy root biomass and tetrandrine yield.

The WPM culture medium was used as the basal medium, and two nitrogen sources, NH_4_NO_3_ (X1) and Ca(NO_3_)_2_ (X2), and one carbon source, sucrose (X3), were selected as the independent variable factors in FFD (2^3^ full factorial design method). The induced hairy roots and tetrandrine production were selected as dependent variables, i.e., response, and analyzed after five weeks of cultivation. The center point was duplicated, and the selected independent variable was represented by coded levels, where −1, 0, and +1 corresponded to low, middle, and high levels, respectively (Table 6). A total of 10 experimental sets were performed, and the impact of the factors was estimated using a first-order polynomial equation. The results obtained from the FFD analysis (Table 1) were used in the next step of the PSA analysis. Two sets of experiments were created based on the two different regression equations obtained for the growth quality of hairy roots and tetrandrine production after FFD analysis. A total of 6 experiments were designed from the regression equation of the growth quality of hairy root biomass. Table 2 displays the steps taken to determine a new unit (the new steep ascent path) and the concentrations of each ingredient in each of the six tests. Similarly, the regression equation of tetrandrine production generated 7 sets of experiments (Table 4). The results of PSA were further used to design the experiments for CCD analysis.

### 3.4. Central Composite Experimental Design

Based on the approximate central point of the response surface in the optimal area determined by PSA, CCD was used to estimate the optimal concentration of each factor within the experimental range. Two groups of experiments, one on the basis of the growth quality of hairy root biomass and the second based on tetrandrine production, were designed based on the results of PSA. The range of new experimental values of factors for hairy root biomass and tetrandrine production is shown in Tables S3 and S4, respectively, and in total, 16 sets of experiments were generated in each group. Multiple regression analysis was used to fit the response (maximum hairy root biomass and tetrandrine production) to a second-order polynomial equation. An analysis of variance (ANOVA) was used to determine the model’s appropriateness. Design Expert 8.0.6 was the software program used to plan and analyze each experiment.

### 3.5. Dry Weight Measurement of Hairy Roots

Wet weight (FW) was calculated by weighing the moisture-absorbed filter paper after the hairy roots were removed from the liquid medium with the aid of sterilized clips. Next, for a whole day, the hairy roots were freeze-dried, and the dry weight (DW) was measured once more.

### 3.6. Analysis of Tetrandrine

The concentration of tetrandrine was examined using high-performance liquid chromatography (HPLC). For sample preparation, a freeze-dried sample (1 g) suspended in methanol (20 mL) was subjected to homogenization at 1000 rpm (HsiangTai HG-202, HsiangTai Machinery Industry Co., Ltd., New Taipei City, Taiwan) followed by 30 min ultrasonication at 40 KHz (DELTA DC100, DELTA, New Taipei City, Taiwan) and soaking overnight in methanol (20 mL). The following day, after centrifugation (4000 rpm, 10 min) (Z383, Hermle, Baden-Württemberg, Germany), the supernatant was collected. For every sample, this process was performed thrice. For HPLC analysis, the combined filtrate of all samples was pressure-dried, suspended in 2 mL of methanol, and filtered using a 0.22 μm filter membrane (Millipore, Burlington, MA, USA). The reverse phase chromatography column (Symmetry Waters RP-C18 column; 5 μm, 4.6 × 250 mm) with an L-2450 PDA-UV detector was used for the separation of the components at ambient temperature. HPLC was performed under isocratic conditions, and a 60:20:20 blend of methanol, acetonitrile, and diethylamine (0.06%) was utilized as the mobile phase, flowing at a rate of 1.0 mL/min. The HPLC-grade tetrandrine was used at the concentration range of 50–1000 mg/L as a standard. The tetrandrine production was obtained using the Equation (5). The chromatograms of standard tetrandrine and extracted tetrandrine are shown in Figure S3.

Tetrandrine production (mg/L) = Tetrandrine obtained after HPLC analysis (mg/g) × hairy root dry weight (g dw/L)(5)

### 3.7. Statistical Analysis

The data were analyzed using ANOVA utilizing the Statistical Analysis System (SAS 9.1), with a probability *p* < 0.05 used to check for the least significant difference (LSD). The HPLC analyses were carried out in triplicates, and the mean ± standard deviation (SD) was used for all data.

## 4. Conclusions

The present study discusses the optimization of medium component concentration for the enhanced production of *S. tetrandra* hair root biomass and tetrandrine. The optimization process included a two-level factorial design, the path of the steepest ascent, and the central composite design. The optimization for biomass production led to a significant improvement in hairy root biomass (9.75 g dw/L), while a separate optimization for tetrandrine production resulted in an enhanced tetrandrine concentration (70.48 mg/L). Though this study demonstrated that tetrandrine production and hairy root biomass responded differently to changes in medium composition, the lack of a direct correlation between these parameters suggests that secondary metabolite accumulation may be triggered under conditions that do not necessarily favor optimal growth. This phenomenon is commonly observed in plant and hairy root cultures, where stress conditions can induce secondary metabolite biosynthesis. These findings provide a basis for future research on tetrandrine production through *S. tetrandra*’s hairy root cultures. Further studies, such as temporal profiling or gene expression analysis of biosynthetic pathways, could elucidate the underlying physiological and molecular mechanisms. Further investigations could target scaling up the process to bioreactors under these optimized conditions. Additionally, the tetrandrine produced should be further analyzed for various biological activities.

## Figures and Tables

**Table 1:** Results of a two-level factorial design for the effect of different concentrations of medium components on *S. tetrandra* hairy root biomass and tetrandrine production.

Experiment	NH_4_NO_3_ (mg/L)	Ca(NO_3_)_2_ (mg/L)	Sucrose (g/L)	Biomass (g dw/L)	Tetrandrine Production (mg/L)
1	400	556	30	7.31 ± 0.09	42.55 ± 1.06
2	600	556	30	7.96 ± 0.04	46.38 ± 2.13
3	400	956	30	7.35± 0.32	48.45 ± 0.02
4	600	956	30	7.50 ± 0.10	58.00 ± 4.02
5	400	556	50	7.35 ± 0.22	20.26 ± 1.26
6	600	556	50	8.22 ± 0.53	24.58 ± 1.90
7	400	956	50	6.95 ± 0.46	26.09 ± 2.60
8	600	956	50	7.86 ± 0.49	33.08 ± 0.09
9	500	756	40	8.92 ± 0.51	38.29 ± 3.32
10	500	756	40	8.72 ± 0.27	37.37 ± 2.22

NH_4_NO_3_: ammonium nitrate, (Ca(NO_3_)_2_: calcium nitrate.

**Table 2:** Experimental design and result of path of steepest ascent analysis of *S. tetrandra* hairy root biomass.

	NH_4_NO_3_ (X_1_, mg/L)	Ca(NO_3_)_2_ (X_2_, mg/L)	Sucrose (X_3_, g/L)	Biomass (g dw/L)
(1) Base point	500	756	40	
(2) Unit	100	200	10	
(3) Slop	0.32	−0.15	0.03	
(4) New unit = (2) × (3)	32	−30	0.3	
Expt. 1	500	756	40	6.89 ± 0.64
Expt. 2	532	726	40.3	7.64 ± 0.46
Expt. 3	564	696	40.6	7.59 ± 0.62
Expt. 4	596	666	40.9	8.27 ± 0.54
Expt. 5	628	636	41.2	9.33 ± 0.27
Expt. 6	660	606	41.5	9.05 ± 0.85

NH_4_NO_3_: ammonium nitrate, (Ca(NO_3_)_2_: calcium nitrate.

**Table 3:** Results of the central composite design for enhanced *S. tetrandra* hairy root biomass.

Experiment	NH_4_NO_3_ (mg/L)	Ca(NO_3_)_2_ (mg/L)	Sucrose (g/L)	Biomass (g dw/L)	
				Experimental Value	Predicted Value
1	628.00	636.00	40.95	7.00 ± 0.02	6.95
2	644.00	621.00	41.35	7.62 ± 0.01	7.54
3	628.00	610.77	41.20	8.32 ± 0.01	8.50
4	644.00	651.00	41.35	9.30 ± 0.02	9.16
5	628.00	636.00	41.20	8.30 ± 0.02	7.73
6	601.09	636.00	41.20	7.23 ± 0.02	7.16
7	612.00	651.00	41.05	8.05 ± 0.01	8.08
8	654.91	636.00	41.20	6.67 ± 0.02	6.83
9	628.00	636.00	41.20	7.17 ± 0.01	7.73
10	628.00	661.23	41.20	10.35 ± 0.03	10.25
11	612.00	621.00	41.35	7.90 ± 0.02	7.74
12	612.00	651.00	41.35	8.77 ± 0.005	8.90
13	644.00	621.00	41.05	7.15 ± 0.02	6.96
14	644.00	651.00	41.05	7.78 ± 0.01	7.89
15	628.00	636.00	41.45	8.00 ± 0.01	8.13
16	612.00	621.00	41.05	7.53 ± 0.02	7.61

NH_4_NO_3_: ammonium nitrate, (Ca(NO_3_)_2_: calcium nitrate.

**Table 4:** Experimental design and result of path of steepest ascent analysis of tetrandrine production from *S. tetrandra* hairy root.

	NH_4_NO_3_ (X_1_, mg/L)	Ca(NO_3_)_2_ (X_2_, mg/L)	Sucrose (X_3_, g/L)	Tetrandrine Production (mg/L)
(1) Base point	500	756	40	
(2) Unit	100	200	10	
(3) Slope	3.09	3.98	−11.42	
(4) Slope/20	0.1545	0.199	−0.571	
(5) New Unit = (2) × (4)	15.45	39.8	−5.71	
Expt. 1	500	756	40	44.52 ± 1.49
Expt. 2	515.45	795.8	34.29	59.68 ± 3.02
Expt. 3	530.9	835.6	28.58	61.98 ± 2.23
Expt. 4	546.35	875.4	22.87	65.22 ± 1.42
Expt. 5	561.8	915.2	17.16	50.12 ± 1.36
Expt. 6	577.25	955	11.45	28.43 ± 2.90
Expt. 7	592.7	994.8	5.74	9.75 ± 0.96

NH_4_NO_3_: ammonium nitrate, (Ca(NO_3_)_2_: calcium nitrate.

**Table 5:** Results of the central composite design for enhanced tetrandrine production from *S. tetrandra* hairy root.

Experiment	NH_4_NO_3_ (mg/L)	Ca(NO_3_)_2_ (mg/L)	Sucrose (g/L)	Tetrandrine Production (mg/L)	
				Experimental Value	Predicted Value
1	546.35	841.93	22.87	67.79 ± 3.00	66.58
2	538.63	895.30	17.16	50.68 ± 1.77	51.02
3	554.07	855.50	28.58	70.02 ± 4.24	70.43
4	546.35	908.87	22.87	60.92 ± 2.70	61.07
5	554.07	895.30	17.16	53.87 ± 2.22	52.38
6	546.35	875.40	22.87	66.29 ± 1.73	66.62
7	546.35	875.40	32.47	66.44 ± 2.23	64.34
8	538.63	895.30	28.58	64.77 ± 2.15	66.21
9	546.35	875.40	13.27	43.08 ± 4.00	44.13
10	546.35	875.40	22.87	66.76 ± 5.18	66.62
11	559.34	875.40	22.87	67.32 ± 1.38	68.70
12	554.07	855.50	17.16	62.28 ± 4.63	61.59
13	533.36	875.40	22.87	64.20 ± 1.20	61.77
14	538.63	855.50	28.58	61.32 ± 1.67	63.55
15	538.63	855.50	17.16	53.34 ± 1.26	54.03
16	554.07	895.30	28.58	66.84 ± 5.19	66.90

NH_4_NO_3_: ammonium nitrate, (Ca(NO_3_)_2_: calcium nitrate.

**Table 6:** Concentrations of three independent factors using a two-level factorial design at the coded level.

Independent Variable	Coded Level		
	−1	0	+1
NH_4_NO_3_ (mg/L)	400	500	600
Ca(NO_3_)_2_ (mg/L)	556	756	956
Sucrose (g/L)	30	40	50

NH_4_NO_3_: ammonium nitrate, (Ca(NO_3_)_2_: calcium nitrate.

## Data Availability

The data generated and analyzed during this work are available from the corresponding author upon request.

## Supplementary Materials

The following supporting information can be downloaded at https://www.mdpi.com/article/10.3390/molecules30081859/s1, Table S1: Analysis of variance results for enhanced *S. tetrandra* hairy root biomass production; Table S2: Analysis of variance results for enhanced tetrandrine production from *S. tetrandra* hairy roots; Table S3: Range of new experimental values of independent variables of central composite design based on growth quality of hairy roots biomass results of the path of steepest ascent; Table S4: Range of new experimental values of independent variables of central composite design based on tetrandrine production results of the path of steepest ascent; Figure S1: Plot between experimental (actual) values versus predicted values for the optimization of hairy root biomass from *S. tetrandra*; Figure S2: Plot between experimental (actual) values versus predicted values for the optimization of tetrandrine production from *S. tetrandra* hairy root biomass; Figure S3: HPLC chromatograms of (a) standard tetrandrine and (b) tetrandrine extracted from *S. tetrandra* hairy roots.
