# Supplementary material for: Media Composition Effects on Hairy Root Biomass and Tetrandrine Production in Stephania tetrandra
Source: Molecules. 2025 Apr 21;30(8):1859. doi: 10.3390/molecules30081859 (PMC12029506; doi:10.3390/molecules30081859)
Supplement: Supplementary file 1 [file molecules-30-01859-s001.zip › molecules-3563444-supplementary.pdf]

Supplementary Material

Media Composition Effects on Hairy Root Biomass and Tetrandrine Production in *Stephania tetrandra*

Chia-Hung Kuo [1,†], Hsuan-Chieh Liu [2,3,†], Parushi Nargotra [1,†], Hsiao-Sung Chan [4], Hsin-Der Shih [5] and Yung-Chuan Liu [2,*]

[1] Department of Seafood Science, National Kaohsiung University of Science and Technology, Kaohsiung 811, Taiwan; kuoch@nkust.edu.tw (C.-H.K.), parushi11nargotra@gmail.com (P.N.)
[2] Department of Chemical Engineering, National Chung Hsing University, Taichung 402, Taiwan; ohohpigjay@gmail.com
[3] Central Region Campus, Industrial Technology Research Institute (ITRI), Nantou 540, Taiwan
[4] Department of Applied Chemistry, Chaoyang University of Technology, Taichung 413, Taiwan; hsiaosungchan@gmail.com
[5] Plant Pathology Division, Taiwan Agricultural Research Institute, Ministry of Agriculture, Taichung 413, Taiwan; tedshih@tari.gov.tw
* Correspondence: ycliu@dragon.nchu.edu.tw; Tel.: +886-4-22853769; Fax: +886-4-22854734
† These authors contributed equally to this work.

Table S1. Analysis of variance results for enhanced *S. tetrandra* hairy root biomass production.

Source	Sum of Squares	df	Mean Square	F-value	p-value	
**Model**	12.30	9	1.37	9.61	0.0061	significant
X_1	0.1318	1	0.1318	0.9276	0.3727	
X_2	3.71	1	3.71	26.07	0.0022	
X_3	1.66	1	1.66	11.68	0.0142	
X_1X_2	0.1058	1	0.1058	0.7444	0.4214	
X_1X_3	0.1013	1	0.1013	0.7124	0.4310	
X_2X_3	0.2450	1	0.2450	1.72	0.2372	
X_1^2	0.6295	1	0.6295	4.43	0.0800	
X_2^2	3.14	1	3.14	22.12	0.0033	
X_3^2	0.0406	1	0.0406	0.2858	0.6122	
**Residual**	0.8527	6	0.1421			
Lack of Fit	0.2143	5	0.0429	0.0671	0.9881	not significant
Pure Error	0.6385	1	0.6385			
**Cor Total**	13.15	15				

X_1: Ammonium nitrate (NH_4NO_3, mg/L), X_2: Calcium nitrate ($Ca(NO_3)_2$, mg/L), X_3: Sucrose (g/L).

Table S2. Analysis of variance results for enhanced tetrandrine production from *S. tetrandra* hairy roots.

Source	Sum of Squares	df	Mean Square	F-value	p-value	
**Model**	824.00	9	91.56	21.58	0.0007	significant
X_1	58.01	1	58.01	13.67	0.0101	
X_2	36.59	1	36.59	8.62	0.0261	
X_3	493.15	1	493.15	116.24	< 0.0001	
X_1X_2	19.16	1	19.16	4.52	0.0777	
X_1X_3	0.2312	1	0.2312	0.0545	0.8232	
X_2X_3	16.07	1	16.07	3.79	0.0995	
X_1^2	2.21	1	2.21	0.5219	0.4972	
X_2^2	9.00	1	9.00	2.12	0.1955	
X_3^2	177.56	1	177.56	41.85	0.0006	
**Residual**	25.46	6	4.24			
Lack of Fit	25.35	5	5.07	45.90	0.1116	not significant
Pure Error	0.1104	1	0.1104			
**Cor Total**	849.46	15				

X_1： Ammonium nitrate (NH_4NO_3, mg/L), X_2： Calcium nitrate ($Ca(NO_3)_2$, mg/L), X_3： Sucrose (g/L).

Table S3. Range of new experimental values of independent variables of central composite design based on growth quality of hairy roots biomass results of the path of steepest ascent.

**Independent variable**	**Symbol**	**Code level**				
		**-1.68**	**-1**	**0**	**+1**	**+1.68**
NH_4NO_3 (mg/L)	X_1	601.09	612	628	644	654.91
$Ca(NO_3)_2$ (mg/L)	X_2	610.77	621	636	651	661.23
Sucrose (g/L)	X_3	40.95	41.05	41.2	41.35	41.45

NH_4NO_3： Ammonium nitrate, ($Ca(NO_3)_2$： Calcium nitrate.

Table S4. Range of new experimental values of independent variables of central composite design based on tetrandrine production results of the path of steepest ascent.

Independent variable	Symbol	Code level				
		**-1.68**	**-1**	**0**	**+1**	**+1.68**
NH_4NO_3 (mg/L)	X_1	533.36	538.63	546.35	554.07	559.34
$Ca(NO_3)_2$ (mg/L)	X_2	841.93	855.5	875.4	895.3	908.87
Sucrose (g/L)	X_3	13.27	17.16	22.87	28.58	32.47

NH_4NO_3： Ammonium nitrate, ($Ca(NO_3)_2$： Calcium nitrate.

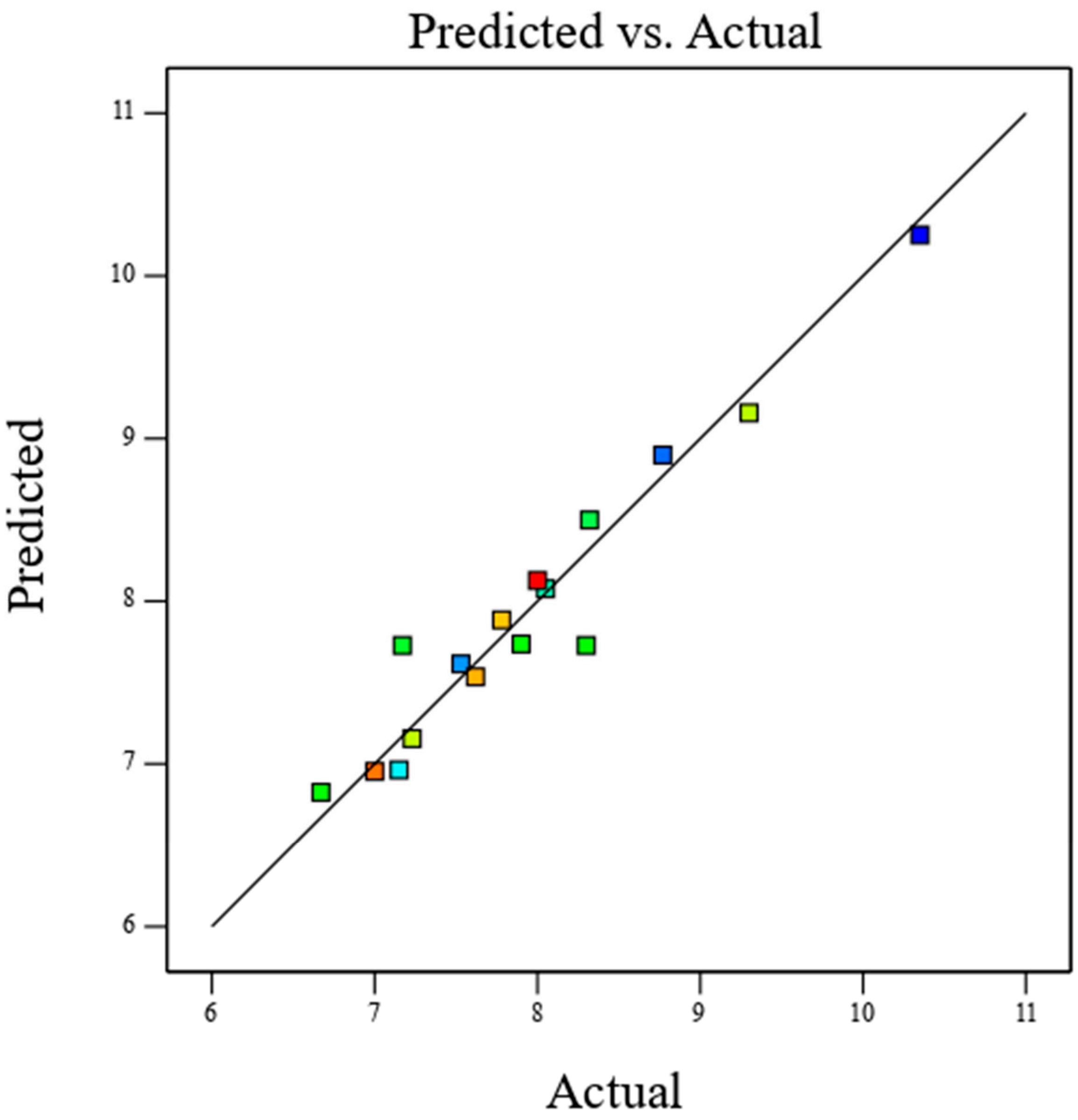

Figure S1. Plot between experimental (actual) values versus predicted values for the optimization of hairy root biomass from *S. tetrandra.*

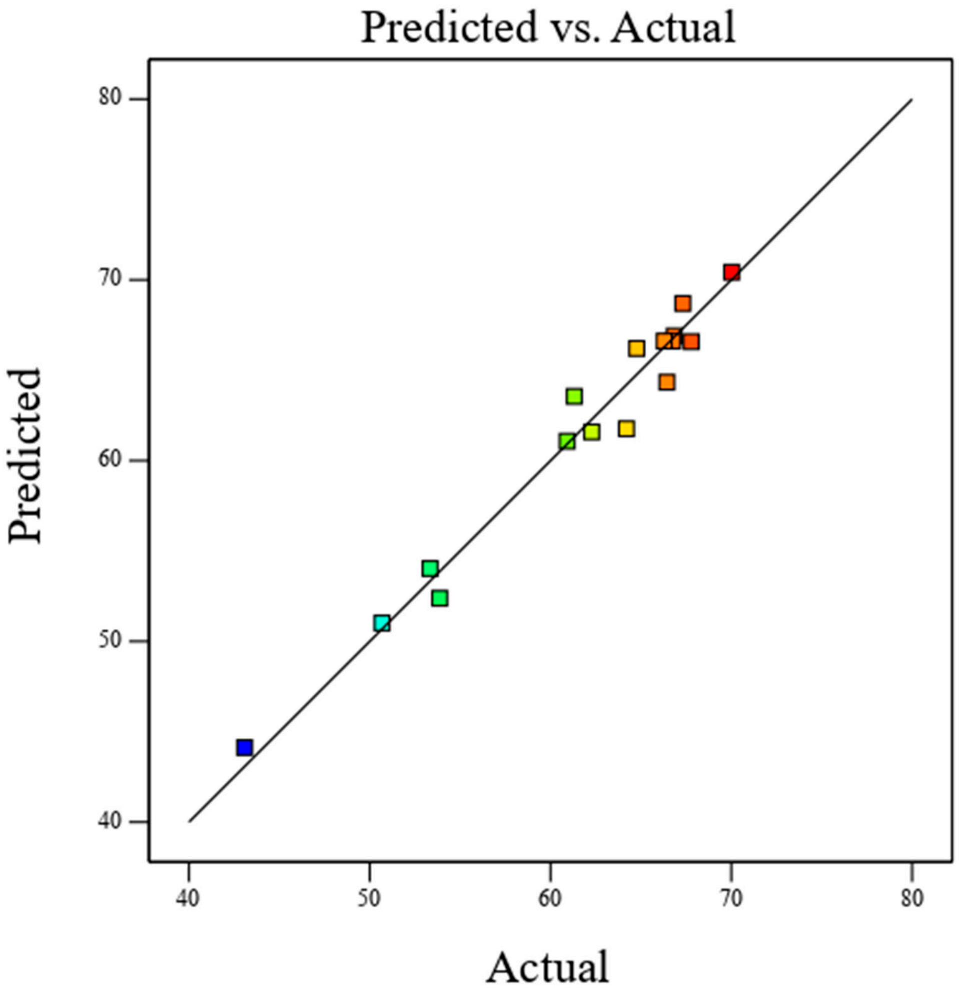

Figure S2. Plot between experimental (actual) values versus predicted values for the optimization of tetrandrine production from *S. tetrandra* hairy root biomass.

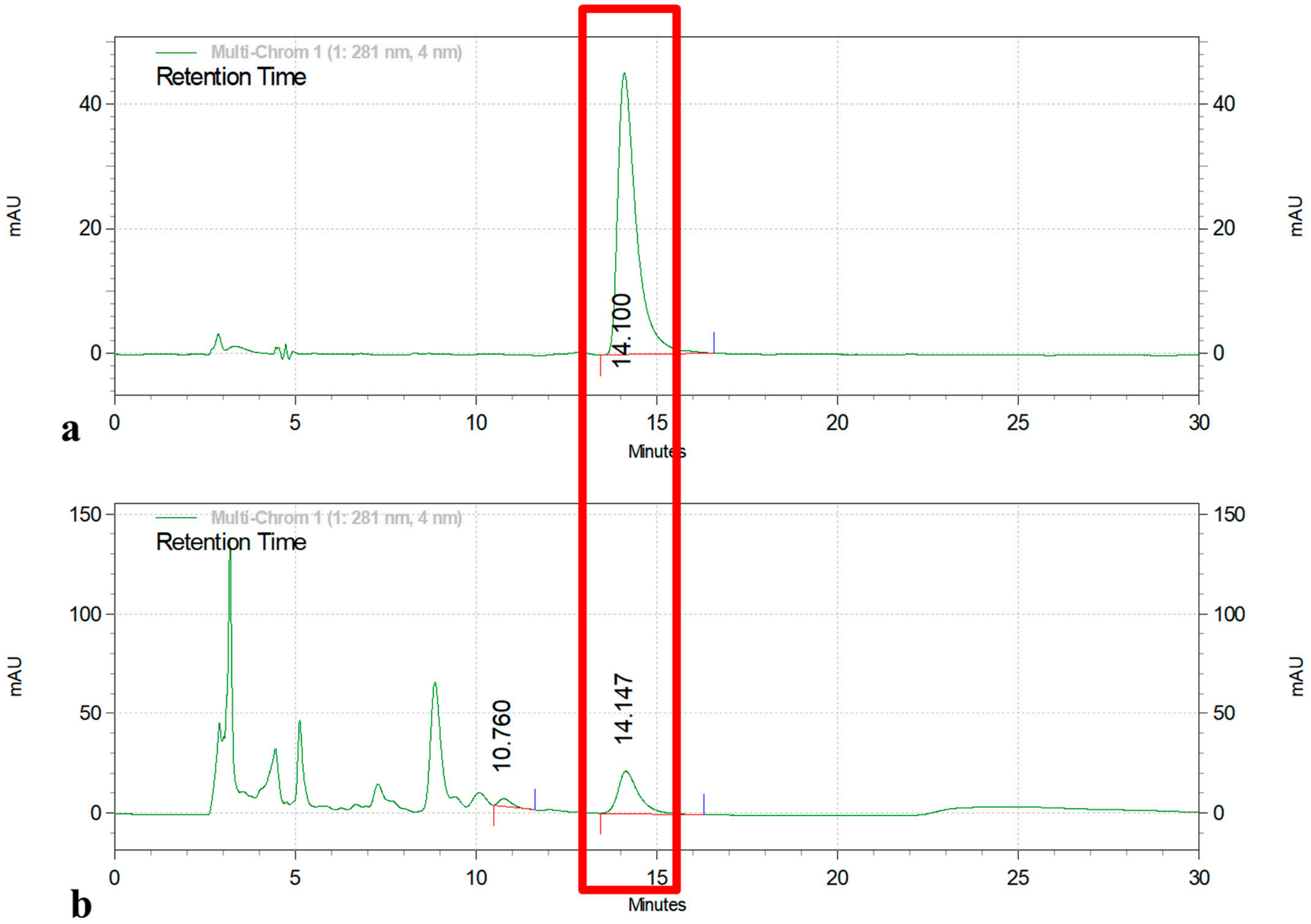

Figure S3. HPLC chromatograms of (a) standard tetrandrine and (b) tetrandrine extracted from *S. tetrandra* hairy roots.
